# Depression and Anxiety Trajectories among Women Who Undergo an Elective Cesarean Section

**DOI:** 10.1371/journal.pone.0086653

**Published:** 2014-01-22

**Authors:** Shu-Yu Kuo, Su-Ru Chen, Ya-Ling Tzeng

**Affiliations:** 1 School of Nursing, College of Nursing, Taipei Medical University, Taipei, Taiwan; 2 School of Nursing, China Medical University, and an Adjunct Supervisor in the Department of Nursing, China Medical University Hospital, Taichung, Taiwan; Harvard Medical School, United States of America

## Abstract

**Background:**

Depression and anxiety are important mood changes in childbearing women. However, changes in depression and anxiety over time in women who undergo an elective cesarean section (CS) have not yet been elucidated. We aimed to characterize the trajectories of depressive and anxiety symptoms, and patterns of co-occurrence, and examined the associated predictors of depression and anxiety courses.

**Methods:**

A prospective longitudinal study of childbearing women (*N* = 139) who underwent a CS was conducted. Depressive and anxiety symptoms were respectively assessed using the Edinburgh Postnatal Depression Scale and State Anxiety Inventory, in the third trimester and at 1 day, 1 week, and 1 and 6 months postpartum.

**Results:**

Group-based modeling identified three distinct trajectories of depressive symptoms: group 1 (low, 30.9%), group 2 (mild, 41.7%), and group 3 (high, 27.3%). Four group trajectories of anxiety symptoms were identified: group 1 (low, 19.4%), group 2 (mild, 44.6%), group 3 (high, 28.8%), and group 4 (very high, 7.2%). Mild symptoms of both depression and anxiety were the most common joint trajectory. Depression trajectories were significantly related to anxiety trajectories (*p*<0.001). Predictors of the joint trajectory included the pre-pregnant body mass index (odds ratio (OR): 2.42, 95% confidence interval (CI): 1.1∼6.3) and a poor sleep score (OR: 3.2, 95% CI: 1.4∼7.3) in the third trimester.

**Conclusions:**

Distinctive trajectories and co-occurrence patterns of depressive and anxiety symptoms were identified. Our findings suggest a need for greater attention to continuous assessment of psychological well-being among women who undergo an elective CS.

## Introduction

Cesarean sections (CSs) continue to rise globally, with an overall rate of 25.7% [1]. The CS rate in the US was reported to be 20.7% in 1996 and rose to 31.1% in 2006 [2], whereas the rate increased from 6% to 46% from 1998 to 2009 in China [3]. Likewise, in Taiwan, the CS rate is rapidly increasing, at 33.9% in 2006 and 37.6% in 2012 [4]. Several studies suggested that the increasing number of women receiving an elective CS without clinical indications may contribute to the rising trend of the CS rate [1]. Factors such as the mother’s mood, anxiety, and fear of birth were associated with women’s preference for a CS [5]. CSs are considered a major surgery, but also an important childbirth event, with both physical and psychological effects on adaptation [6]. Recent studies found that mothers with intense fear of childbirth were more likely to have elective caesarean sections [7], [8] and often experienced elevated depression and anxiety symptoms [9]. However, little is known concerning changes in depressed mood and anxiety over time among women who undergo an elective CS. To this point, few studies have followed women undergoing an elective CS from pregnancy through postpartum period and most have focused on postpartum depression [10].

Depression and anxiety are highly prevalent during the childbirth period [11], [12] and have great impacts on maternal and neonatal outcomes [13], [14]. Elevated depressive and anxiety symptoms were estimated to occur in 37.1% and 54% of women, respectively, during pregnancy [12], while in the postpartum period, rates were 23%∼42.6% for depression [11] and 24.3%∼30.7% for anxiety [15]. Data on the prevalence of depression and anxiety according to mode of delivery is more limited, although a study of postpartum depression found that women who underwent elective CS had higher rate of postpartum depression disorders (32.68%) than those who underwent normal vaginal delivery (17.8%) [16]. A recent review found a positive association between CS and posttraumatic stress symptoms after childbirth [17], whereas elevated posttraumatic stress symptoms were estimated to be in 34% of mothers who underwent emergent CS [18]. Whether such an association exists in mothers with elective CS remains to be investigated. Existing studies of depression and anxiety in perinatal women revealed a pattern of co-occurrence and a certain degree of heterogeneity in the longitudinal course [19], [20], [21]. However, most previous research reported patterns of anxiety and depression using symptom means of the entire sample of women, instead of identifying mothers with varying levels of severity of depression or anxiety across time. This is of concern, in that identifying distinct groups with depression and anxiety is useful for making more-targeted efforts in preventing and intervening in both conditions. A trajectory analysis can help elucidate sources of heterogeneity within a sample of individuals [22]. Such a trajectory approach can identify women with similar degrees of depression or anxiety and then classify them into subgroups according to their original level and changes over time in depressive and anxiety symptoms. Nevertheless, whether distinct longitudinal trajectories of depression and anxiety are present and the relation between depression and anxiety trajectories in women who undergo a CS remain unclear.

A number of factors may predict different depressive and anxiety symptoms in women undergoing childbirth. Among them, modifiable factors such as body weight and sleep quality are of particular clinical importance [11], [23]. Obese women tend to experience higher levels of depressive and anxiety symptoms after delivery [24], [25]. Sleep difficulties during late pregnancy are associated with moods and anxiety in the perinatal period [26], [27]. Most research so far mainly focused on correlates of depressive or anxiety symptoms, predictors of depressive and anxiety symptoms trajectories, and the co-occurrence patterns in CS mothers are unknown.

In this study, we hypothesized that distinct trajectories and co-occurrence patterns of depressive and anxiety symptoms ranging from low to high levels of symptoms could be detected. We conducted a prospective, longitudinal study of CS women with five time-point measurements to examine temporal changes in depression and anxiety. The purposes were to (a) identify distinct subgroups of women following different depression and anxiety trajectories, and (b) examine how the depression trajectory is related to anxiety patterns and associated predictors of distinct depression and anxiety trajectories from late pregnancy to 6 months postpartum among women undergoing an elective CS.

## Methods

### Participants

The participants in this study were pregnant women who received antenatal care in the clinic of a university hospital in Taiwan. Eligible participants included pregnant women aged ≥20 years who considered having an elective CS. Women with perinatal complications or a chronic medical illness were excluded. In total, 150 eligible women were approached for participation and provided informed consent. Among them, 11 women (14%) only completed the baseline information and were unable to participate the follow-up surveys due to a lack of time or family concerns. In total, 139 women were included in this study.

### Procedures

A prospective longitudinal design with five assessments was used. Participating women completed the initial assessment in their third trimester at the antenatal clinic. The second and third assessments were respectively completed on postpartum day 1 and week 1 before hospital discharge. The fourth and fifth assessments were carried out during a home visit at 1 and 6 months postpartum. The study protocol received approval by the institutional review board of China Medical University Hospital, Taiwan.

### Measures

#### Depressive symptoms

Depressive symptoms were measured by the Taiwanese version of the Edinburgh Postnatal Depression Scale (EPDS) [28]. It is a 10-item self-reported questionnaire with a 4-point scale that ranges from 0 (“no”) to 3 (“most of the time”), with a total score ranging 0∼30. A higher score represents a higher level of depression. The suggested cutoff of 12/13 was used to detect probable cases of clinical depression, with a respective sensitivity and specificity of 83% and 89% [29]. Cronbach’s alpha reliability of the Taiwanese version of EPDS was 0.87 [28]. In our study, Cronbach’s alpha of the EPDS ranged 0.82∼0.86.

#### Anxiety symptoms

The Taiwanese version of the State Anxiety Inventory (SAI) was used to measure anxiety levels. It contains 20 items rated on a scale of 1 (not at all) to 4 (very much so), with an overall score ranging 20∼80. A higher score indicates higher anxiety. The SAI was shown to have good psychometric properties among childbearing women, with an internal reliability of 0.92 [30]. A score ≥45 is considered indicative of a high-anxiety state among pregnant women [31]. The Taiwanese version of the SAI showed good internal consistency (0.90) [32]; in the present study, Cronbach’s alpha of the SAI was 0.89∼0.91.

#### Sleep quality

Sleep quality and disturbances were measured by the Taiwanese version of the Pittsburgh Sleep Quality Index (PSQI). The 19-item PSQI is a self-reported questionnaire and generates seven components scores (0∼3) to produce a global score ranging from 0 (good sleep) to 21 (very poor sleep). The Taiwanese version of the PSQI has been validated with good internal reliability and test-retest reliability [33]. A global score of >8, which was suggested as an appropriate cutoff score in clinical populations, is used to identify sleep difficulties [34]. Cronbach’s alpha of the PSQI in childbearing women was 0.73 [35]; in our study, the range of Cronbach’s alpha of the PSQI was 0.70∼0.78.

#### Demographic features and the health status

A structured questionnaire was used to investigate predictors for changes in depression and anxiety over time. The main variables included demographic features (age and educational attainment), physiologic variables (parity, prenatal exercise, and pre-pregnancy body mass index (BMI)), and situational variables (employment status and use of patient-controlled analgesics (PCAs)) that were shown to be associated with depression or anxiety [11], [15].

### Statistical Analysis

Descriptive statistics were used to describe characteristic of participants at the baseline. Established cutoff scores were used to identify elevated levels of depression or anxiety that were of clinical relevance. To identify distinct groups of women with similar patterns of change in depressive and anxiety symptoms over time, a group-based trajectory analysis was applied based on mother’s EPDS or SAI score from their third trimester until postpartum 6 months [36]. Group-based trajectory modeling is a person-centered analysis which is designed to estimates growth curves for each individual and then classified individuals with similar growth curves into different pattern of trajectory groups. Such analyses allow researchers to examine the distinct pattern of trajectories of maternal depression [37] or other problem behaviors [38]. We applied PROC TRAJ in SAS (version 9.2 SAS Institute, Cary, NC) [39], which optimally uses available data for a maximum-likelihood estimation. Based on the Bayesian information criterion (BIC) value, optimal trajectory groups were determined [36]. The best-fitting model was selected based on the smallest absolute Bayesian Information Criterion (BIC) value. Individuals were classified into different depressive symptoms or anxiety trajectory groups according to the highest posterior probabilities of group membership.

Initially, trajectory groups for depression and anxiety were separately identified, and then the joint probability of group membership in depression and anxiety was estimated. Joint trajectory procedures allowed us to determine the interrelationship of the two types of distinct but related symptoms. Joint probabilities of the joint model describe the proportion of women estimated to simultaneously be experiencing trajectories of depression and anxiety, while the transition probabilities of the two symptoms represent the percentage of participants following different combinations of depression and anxiety trajectories [36]. Associations between baseline characteristics and depression or anxiety trajectories were tested using a logistic regression analysis.

## Results

The majority of participants were multipara (61.9%), had at least some college education (73.4%), and were employed (64%) (Table 1). More than half of the women (64%) had not engaged in prenatal exercises; 66.2% of women reported a planned pregnancy; and 85.6% had used PCAs after the CS. Overall, the mean age of participants was 33.6 (standard deviation (SD) = 3.8) years, and mean gestational age at delivery was 37.3 (SD = 2.0) weeks. The BMI before pregnancy of participants was 22.6 (SD = 4.0) kg/m^2^, and average birth weight of the newborns was 3363.4 (SD = 678.3) g.

**Table 1:** Baseline Characteristic of Participants (*N* = 139).

Variable	*n*	(%)	
Parity			
Primipara	53	(38.1)	
Multipara	86	(61.9)	
Educational level			
High school or below	37	(26.6)	
College or above	102	(73.4)	
Prenatal employment			
No	50	(36.0)	
Yes	89	(64.0)	
Prenatal exercise			
No	89	(64.0)	
Yes	50	(36.0)	
Planned pregnancy			
No	47	(33.8)	
Yes	92	(66.2)	
Use of patient-controlled analgesics			
No	20	(14.4)	
Yes	119	(85.6)	
PSQI			
≤8	66	(47.5)	
>8	73	(52.5)	
	***N***	***Mean***	***(SD)***
Age (years)	139	33.6	(3.8)
Gestational age (weeks)	139	37.3	(2.0)
Pre-pregnancy BMI (kg/m^2^)	139	22.6	(4.0)
Birthweight of newborn (g)	139	3363.4	(678.3)

Note: SD, standard deviation; PSQI = Pittsburgh Sleep Quality Index; BMI = body-mass index.

Of the 139 eligible participants, all of the participants (100%) completed four assessments, and 102 (73%) completed all five assessments. Those who participated in all five time points and those who dropped out at postpartum 6 months did not significantly differ in the mean scores of depression or anxiety at times 1, 2, 3, or 4 (*p* = 0.18∼0.99). In general, the demographic characteristics (i.e., age, parity, educational level, prenatal employment, and prenatal exercise) of the two groups were similar (*p* = 0.14∼0.90).

The mean depression score was higher in late pregnancy (mean = 9.7, SD = 5.1) then gradually decreased to 7.6 at 6 months postpartum. The mean anxiety scores were relatively stable over time, with a peak on postpartum day 1 of 42.2 (SD = 8.9), with the lowest score at 6 months postpartum (40, SD = 9.5) (Table 2). Depression was less prevalent than anxiety, and both peaked at 4 weeks postpartum. The prevalence of depression was 28.1% in late pregnancy. The rate respectively decreased to 25.2% and 26.6% at day 1 and week 1 postpartum, but increased again to 28.8% at postpartum 4 weeks and then dropped to 20.8%. For anxiety, the prevalence was 30.2% in the third trimester, remained stable (29.5%∼30.2%) to week 1 postpartum, then increased to 37.4% at week 4 postpartum, and dropped to 30.4% at 6 months postpartum.

**Table 2:** Edinburgh Postnatal Depression Scale (EPDS) and State Anxiety Inventory (SAI) at Each Assessment.

	36 weeks of pregnancy		1 day postpartum		1 week postpartum		4 weeks postpartum		6 months postpartum	
Edinburgh Postnatal Depression Scale	*n* = 139		*n* = 139		*n* = 139		*n* = 139		*n* = 101	
Mean (SD)	9.7	(5.1)	9.2	(5.1)	9.2	(5.3)	8.9	(6.1)	7.6	(5.4)
No. (%) depressed (EPDS >12)	39	(28.1)	35	(25.2)	37	(26.6)	40	(28.8)	21	(20.8)
State Anxiety Inventory	*n* = 139		*n* = 139		*n* = 139		*n* = 139		*n* = 102	
Mean (SD)	41.0	(9.0)	42.2	(8.9)	41.7	(10.0)	42.0	(11.0)	40.0	(9.5)
No. (%) with anxiety (SAI >45)	42	(30.2)	41	(29.5)	42	(30.2)	52	(37.4)	31	(30.4)

Note: SD, standard deviation; Range of EPDS score = 0–28; Range of SAI score = 20–78.

### Trajectory Groups of Depressive and Anxiety Symptoms

**Figure 1:**
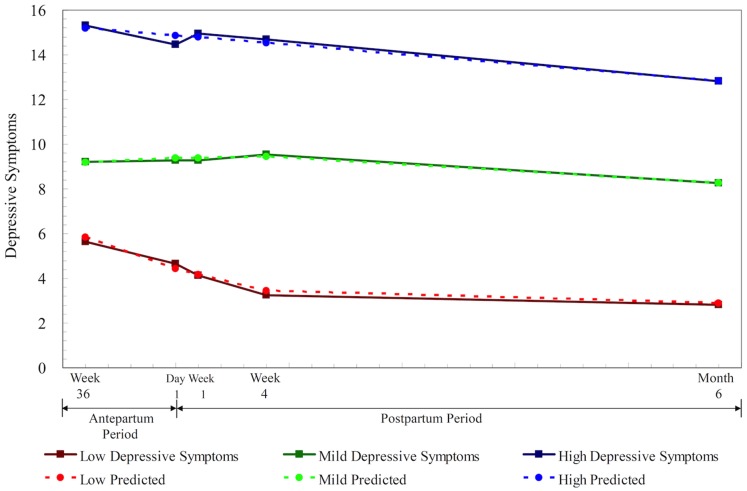
Actual versus predicted trajectories of depressive symptoms among women who underwent a cesarean delivery (*N* = 139): low depressive symptoms (30.9%), mild depressive symptoms (41.7%), and high depressive symptoms (27.3%).

We identified three depression trajectory groups as the best fitting model based on the lowest absolute BIC (−1908.08). As seen Figure 1, 30.9% of mothers (*n* = 43) followed a low depression trajectory, 41.7% (*n* = 58) followed a mild depression trajectory, and 27.3% (*n* = 38) followed a high and stable depression trajectory. The average posterior probability of the depression trajectories was relatively high ranging between 0.87 (standard error (SE) = 0.13) and 0.91 (SE = 0.12), indicating adequate model fitting based on the suggested criteria of an average posterior probability of ≥0.70 [40].

**Figure 2:**
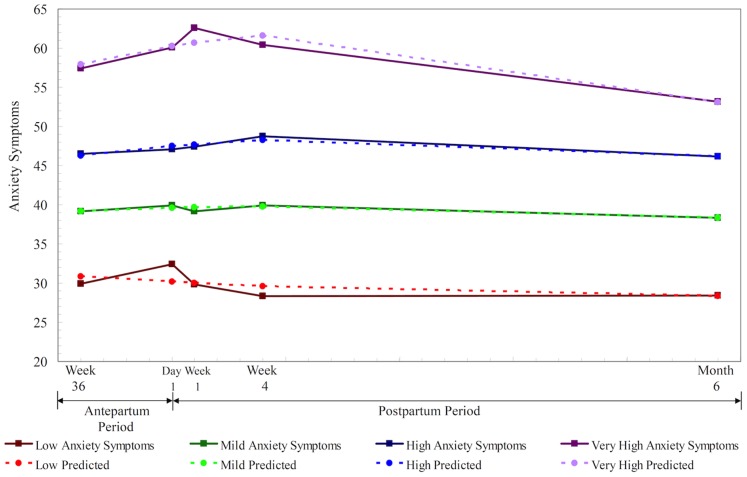
Actual versus predicted trajectories of anxiety symptoms among women who underwent a cesarean delivery (*N* = 139): low anxiety symptoms (19.4%), mild anxiety symptoms (44.6%), high anxiety symptoms (28.8%), and very high anxiety symptoms (7.2%).

For anxiety symptoms, four anxiety groups were identified (BIC = −2260.19): 19.4% (*n* = 27) of mothers followed a low anxiety trajectory; 44.6% (*n* = 62) a mild anxiety trajectory; 28.8% (*n* = 40) a high anxiety trajectory; and 7.2% (*n* = 10) a very high trajectory (Fig. 2). The average posterior probability of the anxiety trajectories was between 0.87 (SE = 0.16) and 0.97 (SE = 0.08), which provided further support for the four-group model.

### Joint Analysis of Depression and Anxiety Trajectories

The first part of table 3 presents the proportion of women in each joint trajectory group. The highest joint probability involved women with mild levels of both depressive and anxiety symptoms (42.9%), followed by women with low symptoms severity of both (24.9%), third was 23% of women with high levels of both depressive and anxiety symptoms, and fourth included women with high depressive symptoms and very high anxiety symptoms (6.9%) (Table 3).

**Table 3:** Joint and Transitional Probabilities (%) of Classification in the Anxiety and Depressive Symptoms Trajectories (*N* = 139).

	Depressive symptoms			
Anxiety symptoms	Low		Mild	High
Probability of joint trajectory of group membership				
Low	(1) 24.9		0	0.7
Mild	0.7		(2)42.9	0
High	0.9		0	(3)23
Very high	0		0	(4)6.9
Probability of anxiety symptoms based on depressive symptoms				
Low	94.1		0	2.4
Mild	2.6		100	0
High	3.3		0	75.0
Very high	0		0	22.6
Probability of depressive symptoms based on anxiety symptoms				
Low		97.2	0	2.8
Mild		1.6	98.4	0
High		3.7	0	96.3
Very high		0	0	100

Women’s probabilities of membership in each anxiety group based on their depression trajectory group are shown in the second part of table 3. Women with low symptom severity of depression were more likely (94.1%) to be in the low symptom severity class of anxiety, while those with mild levels of depression were almost certain (100%) to have mild levels of anxiety. Those with high symptom severity of depression were more likely to have either high (75%) or very high levels of anxiety (22.6%). Similar findings of women’s probabilities of being in a certain depression trajectory group given their anxiety group membership are shown in the third part of table 3. Together, results of the transition probabilities indicated that women tended to follow the same symptom severity of both depressive and anxiety symptoms. Cross-classification results also confirmed that depressive symptom and anxiety symptom trajectories were highly associated (Chi-squared = 258.3, d.f. = 6, *p*<0.001).

### Baseline Variables Associated with Depression, Anxiety, and Joint Trajectories

Logistic regression analyses examined how baseline variables were associated with depression, anxiety, and the joint trajectory class, using ‘low’ and ‘mild’ as the reference categories. The univariate analyses results showed that women with a higher BMI (≥24 kg/m^2^) before pregnancy or with sleep difficulties (PSQI >8) in the third trimester were significantly more likely to have high levels of depression and anxiety, and joint trajectories (Table 4). Meanwhile, women using PCAs after the CS had decreased odds of being in a high-depression trajectory. We used asterisks in the *n* (%) column in Table 4 to indicate the significance level of the crude odds ratios (ORs). In the multivariate analyses, the adjusted ORs of these variables remained significant for the high-depression trajectory class, high/very high-anxiety trajectory class, and high/very high joint trajectory class, except that the association between the pre-pregnancy BMI and high depression class was non-significant. Neither the age, parity, nor educational level was associated with any high-symptom trajectory.

**Table 4:** Relationship of Baseline Characteristics with Depression Trajectories, Anxiety Trajectories, and Joint Trajectories.

	Depression trajectory					Anxiety trajectory					Joint trajectory				
	Low/Mild		High			Low/Mild		High/Very high			Low/Mild		High/Very high		
Variable	*n*	(%)^a^	*n*	(%)^a^	aOR (95% CI)^b^	*n*	(%)^a^	*n*	(%)^a^	aOR (95% CI)^b^	*n*	(%)^a^	*n*	(%)^a^	aOR (95% CI)^b^
Age (years)															
≤30	24	(23.8)	7	(18.4)	1	22	(24.7)	9	(18.0)	1	23	(23.7)	8	(19.1)	1
>30	77	(76.2)	31	(81.6)	1.83 (0.6?5.5)	67	(75.3)	41	(82.0)	1.45 (0.6?3.8)	74	(76.3)	34	(80.9)	1.28 (0.5?3.5)
Parity															
Primipara	38	(37.6)	15	(39.5)	1	37	(41.6)	16	(32.0)	1	39	(40.2)	14	(33.3)	1
Multipara	63	(62.4)	23	(60.5)	0.72 (0.3∼1.7)	52	(58.4)	34	(68.0)	1.66 (0.7∼3.8)	58	(59.8)	28	(66.7)	1.35 (0.6?3.1)
Educational level															
High school or below	23	(22.8)	14	(36.8)	1	23	(25.8)	14	(28.0)	1	25	(25.8)	12	(28.6)	1
College or above	78	(77.2)	24	(63.2)	0.53 (0.2∼1.3)	66	(74.2)	36	(72.0)	0.98 (0.4∼2.4)	72	(74.2)	30	(71.4)	1.11 (0.4∼2.8)
Prepregnancy BMI (kg/m^2^)															
<18.5	13	(12.9)	1	(2.6)	0.20 (0.02?1.7)	10	(11.2)	4	(8.0)	0.83 (0.2?3.1)	12	(12.4)	2	(4.8)	0.44 (0.1∼2.2)
18.5∼24	68	(67.3)	11	(57.9)	1	63	(70.8	27	(54.0)	1	67	(69.1)	23	(54.8)	1
≥24	20	(19.8)	15	(39.5)*	2.24 (0.9?5.5)	16	(17.0)	19	(38.0)*	2.43 (1.1?5.9)*	18	(18.5)	17	(40.4)*	2.42 (1.1?6.3)*
Use of PCAs															
No	11	(10.9)	9	(23.7)	1	13	(14.6)	7	(14.0)	1	12	(12.4)	8	(19.1)	1
Yes	90	(89.1)	29	(76.3)*	0.32 (0.1?0.96)*	76	(85.4)	43	(86.0)	1.06 (0.4?3.1)	85	(87.6)	34	(80.9)	0.5 (0.2?1.5)
PSQI															
≤8	55	(54.5)	11	(29.0)	1	53	(59.6)	13	(26.0)	1	54	(55.3)	12	(28.6)	1
>8	46	(45.5)	27	(71.0)	3.15 (1.3?7.4)*	36	(40.4)	37	(74.0)**	4.53 (2.0?10.1)*	43	(44.7)	30	(71.3)**	3.2 (1.4?7.3)*

^a^ The significance level of the univariate odds ratio (OR) is indicated by asterisks in this column if significant.

^b^ Adjusted OR (aOR) and its 95% confidence interval (CI), obtained with statistical adjustment for all the variables listed in this table.

*p*<0.05; **p<0.01.

PSQI, Pittsburgh Sleep Quality Index; PCAs, patient-controlled analgesics; BMI, body-mass index.

## Discussion

The current study is the first to investigate the pattern of changes in symptoms of depression and anxiety among women undergoing an elective CS. The person-oriented trajectory approach revealed distinct patterns in anxiety and depressive symptoms that would not have been detected by describing mean-level changes across time [36]. We found distinct subgroups of depression and anxiety trajectories and evidence for the co-occurrence between the two trajectories. Being overweight before pregnancy and having sleep difficulties in the third trimester predicted mothers having long-term levels of depressive and anxiety symptoms, after adjusting for potential confounders.

A CS involves major physical and psychological adaptation as mothers experience the transition to parenthood during pregnancy and after childbirth [6]. In our study, both depression and anxiety were common, at 25.2%∼28% for depressive symptoms and 29.5%∼37.4% for anxiety symptoms. Specifically, more than one-third of CS mothers experienced elevated levels of anxiety, which were higher than those of depression, implying assessments of psychological disturbances that focus on depression are insufficient. Our findings that anxiety was more prevalent than depression are in line with current evidence that symptoms of anxiety are common during perinatal period [12], [41]. In their study of women requesting CS, Willund and colleagues found that women often reported anxiety concerning lack of support during labor, loss of control, and fetal injury/death [42]. Mothers might have had increased anxiety as a result of expecting that surgery was planned for birth and worried about the surgery procedure, such as anesthesia, of being cut, and the physical environment of the operating room [6]. Possible explanations for the elevated levels of depression and anxiety symptoms could be that the significant changes in physiological functions during perinatal stage, such as hormonal dysreguation [43]. In addition, recent studies showed that the high fear of childbirth during pregnancy was common in women with elective CS [44]. Fear of child birth, often associated with anxiety (OR: 4.8, 95% CI: 4.1∼5.7) [9] and depressed mood (OR: 8.4, 95% CI: 4.8∼14.7) [45], and become a common cause of preference for CS [46]. Furthermore, our study covers an important time period during the transition from pregnancy to motherhood. Adaptation to motherhood in postpartum period was considered as a stressful period and was often linked to the negative psychological state, such as high depression and anxiety [20], [21]. Intriguingly, a high prevalence of depression or anxiety was found at week 4 postpartum in the sample as a whole, which agreed with previous findings that the peak prevalence of postpartum depression or anxiety occurs during 1 month after delivery. Britton *et al.* found high anxiety observed in 30.7% of women at 1 month postpartum compared to 24.3% before discharge from the hospital [15], [47]. In a population-based study of 2178 women, Lau *at al.* found that 8.7% of women had severe depressive symptoms (EPDS >14) at 6 weeks postpartum compared to 7.8% in the third trimester [15], [47]. One possible explanation may be the elevated posttramatic stress symptoms after childbirth may contribute to high depression and anxiety at 4 weeks postpartum [48]. Mothers often experienced great stress exposure during postpartum and may link to depression and anxiety accompany with the process of maternal adaptation [11]. In addition, greater demands (i.e., infant care and additional maternal responsibilities such as returning to work) for some women might account for the reported peak prevalence found at 4 weeks postpartum [15], [47]. Indeed, over 60% of mothers in our study were employed full-time and usually returned to work at 6 weeks postpartum. Although this study used self-report measures for assessment of depressed mood and anxiety, the EPDS and SAI are regularly used in studies with childbirth women and have been adequately validated against clinical diagnostic interview [29], [49]. Hence, the depression and anxiety trajectories identified in the present study are less likely to be due to the EPDS and SAI scoring, but rather are an accurate reflection of the severity of mother’s depressive and anxiety symptoms.

This study clearly identified distinguishable trajectories for depressive and anxiety symptoms in CS women. Consistent with past studies on maternal symptoms over time during childbearing [50], [51], [52] or in the childrearing stage [53], our results indicated heterogeneity in the development of depressive and anxiety symptoms. Identifying subgroups with varying symptoms severity is vital for targeting at-risk women [6]. Our findings showed that a substantial proportion of CS mothers reported high depressive symptoms (27.3%, EDPS >12) or high/very high anxiety (36%; SAI >45) at all assessments, indicating that mothers with high levels of depressive or anxiety symptoms could be identified with early screening. In addition, our findings revealed the static nature of the symptoms trajectory for both depressive and anxiety symptoms, that were consistent with prior longitudinal studies where stable patterns of depression and anxiety were observed in perinatal women [20], [27]. Taken together, the clinical implications of these results highlight the importance of assessing the mood or anxiety status both during pregnancy and postpartum, rather than just in the postpartum period.

The joint analysis of depressive and anxiety symptoms demonstrated a moderate longitudinal association between these two symptom types. Four patterns of comorbid depressive and anxiety symptoms developed, suggesting that depression and anxiety tend to cluster together and persist over time. The pattern of co-occurrence supported women with high depressive symptoms being more likely to also have high symptoms of anxiety than being in the mild or low symptoms clusters. Indeed, prior research showed a concurrent association or prospective link between depression and anxiety through pregnancy and the early postpartum period [20], [21], [23], [31], [54]. Recent studies demonstrated that comorbid maternal depression and anxiety were found to be linked with subsequent perinatal outcomes [55]. However, only a few studies assessed depressive symptoms and anxiety at more than one time point. Further study is thus needed to investigate the association between the long-term co-occurrence patterns of both symptoms and perinatal outcomes.

Baseline predictors for both the depression and anxiety trajectories were similar, suggesting consistent features of these variables in predicting long-term depression and anxiety. A higher BMI before pregnancy and a poor sleep score in the late trimester were found to be significant predictors for high symptoms of depression and anxiety trajectories in CS women. This is congruous with other studies in Western populations [11], [56] that found a significant association between postpartum depression and being obese before the pregnancy. For example, in a recent cohort study conducted on 1053 women, women with pre-pregnancy obesity were more likely to have elevated levels of depressive symptoms (EPDS ≥12) at 6∼8 weeks postpartum (OR: 2.87, 95% CI: 1.21∼6.81) [15], [47]. Similarly, Carter *et al.* [24] found that overweight women were associated with elevated depressive and anxiety symptoms at 4 months postpartum. Several hypotheses were suggested to underlie the link between a high BMI and depression, such as dysregulation of the hypothalamic-pituitary-adrenal axis and immunologic system, eating behaviors, and reduced support [57]. Future research including objective measurements of both physiological and behavioral indicators is required to better understand the possible mechanisms accounting for the association between obesity and trajectories of depression and anxiety.

Again, our finding of sleep difficulties in the third trimester predicting high levels of depressive and anxiety trajectories concurs with a recent review which reported that sleep difficulties during pregnancy predict subsequent depression [58]. Specifically, women who reported greater sleep disturbances in the last month of pregnancy experienced high depressive symptoms at 3 months postpartum [59]. An objective sleep study showed that CS mothers tended to have an average of 4 h total sleep time with 34% waking after sleep onset in the first week postpartum, indicating sleep problems existed in the postpartum period in women after a CS [60]. These findings highlight the importance of assessing for sleep difficulties during pregnancy and postpartum for timely identification and interventions to aid the mother in adjusting to a CS.

Moreover, women who used PCAs after a CS were less likely to be in the high symptoms group of depression trajectories which is similar to a previous finding that mothers who received epidural blockage during delivery had decreased odds of depression in the first postpartum week [61]. Several studies reported that pain and depressive symptoms were closely associated: as levels of pain increased, depressive symptoms became greater [62]. Thus, women use PCAs might experience less pain after a CS, which then might affect their mood changes in the postpartum period.

Several limitations need to be taken into account when interpreting our results. First, depression and anxiety were both assessed by well-standardized self-reported questionnaires, instead of using a clinical diagnosis. However, the dimensional approach is useful for hypothesis testing [63], i.e., identifying women with various symptom severities in this study. Second, the findings are limited to CS mothers without perinatal complications or a chronic medical history and may decrease the ability to generalize of our results to other populations. Third, our sample size was insufficiently large to separately identify predictors for high and very high trajectories. Future research with a larger sample size could test whether profiles of predictors were similar for the high and very high trajectories. Finally, the data were obtained from women undergoing an elective CS, thus lacking of a control group. Future studies include comparison groups such as women with normal vaginal birth would help to add information on the trajectories studied.

In conclusion, by prospectively assessing CS women at multiple time points, we found evidence of distinct longitudinal trajectories of depressive and anxiety symptoms. In adding to the existing literature, this study provides a description of the development of a depressed mood and anxiety over the perinatal period and uncovered heterogeneity and co-occurrence patterns for both symptoms. Additional research is needed to investigate how depression and anxiety trajectories impact maternal and neonatal outcomes and determine optimal prevention strategies and interventions for these CS women. Furthermore, our findings suggest a need for greater attention to continuous assessments of psychological well-being among women who undergo an elective CS.
